# Acquired von Willebrand Disease Associated with Monoclonal Gammopathy of Unknown Significance

**DOI:** 10.1155/2017/9295780

**Published:** 2017-11-01

**Authors:** Sijan Basnet, Catherine Lin, Rashmi Dhital, Izza Mir, Elan Mohanty, Biswaraj Tharu, Sushil Ghimire, Dilli Ram Poudel

**Affiliations:** ^1^Department of Medicine, Reading Hospital, 420 S. Fifth Avenue, West Reading, PA 19611, USA; ^2^Maharajgunj Medical Campus, Tribhuvan University, Kathmandu, Nepal

## Abstract

We present a case of a 79-year-old male who presented with retroperitoneal hematoma a week after motor vehicle accident. Prior history and family history of bleeding were nonsignificant. His activated partial thromboplastin time was found to be prolonged in the emergency department. Further workup with coagulation studies showed decreased factor VIII, vWF antigen, and vWF:ristocetin cofactor assay, and negative Bethesda assay, indicating acquired von Willebrand disease. Immunofluorescence to find an underlying etiology was suggestive of MGUS. Management of AvWD depends on controlling active bleeding and treating the underlying cause. He was treated with factor VIII, haemate-p, rituximab, two cycles of IVIg, and three weeks of oral steroids.

## 1. Introduction

Acquired von Willebrand disease (AvWD) is a rare bleeding disorder [1, 2]. It is characterized by hemorrhagic events later in life without personal or family history of bleeding events [3]. A number of acquired conditions have been associated with AvWD [4]. We report a patient diagnosed with acquired von Willebrand disease secondary to monoclonal gammopathy with unknown significance after initial presentation with retroperitoneal hematoma.

## 2. Case Description

A 79-year-old man presented to the emergency department with severe left hip and groin pain with overlying hematoma. He had a prior admission one week before with arrhythmia-induced syncope leading to a motor vehicle accident. He received a dual-chamber automatic implantable cardioverter-defibrillator after inducible ventricular fibrillation/ventricular tachycardia during electrophysiology study. CT scan at the time was unremarkable except for a 2 cm area of suspected hematoma in the left trochanteric region. His past medical history and medications were not significant except for aspirin use. The patient denied significant prior or family history of bleeding or easy bruising. He had undergone an appendectomy and bilateral hip replacements in the past without significant bleeding.

**Figure 1:**
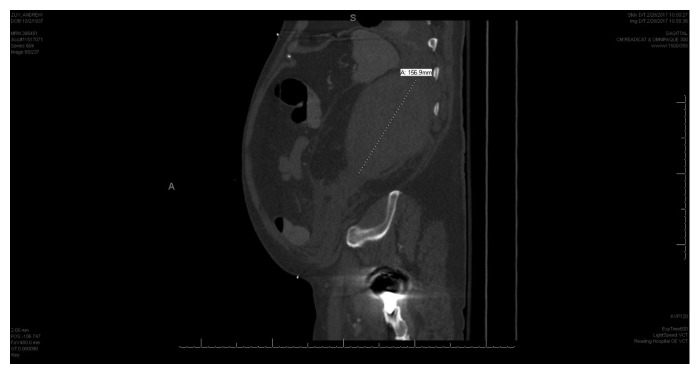
CT scan of the abdomen with retroperitoneal hematoma.

The patient was hypotensive on presentation. His hemoglobin had dropped from 11.7 g/dl to 6.2 g/dl within one week. CT scan of his chest/abdomen/pelvis revealed a large left retroperitoneal hematoma measuring approximately 14.2 × 6.8 × 14.1 cm (Figure 1). The patient's PTT was 48 s, which increased from 38 s a week ago (Table 1). His prothrombin time was 13.9 s with INR of 1.1. His white blood count and platelet count were normal. Serum fibrinogen, fibrin degradation products and d-dimer, and liver function tests were normal. His factor VIII activity was 12%, and von Willebrand factor antigen was 4%. vWF had normal multimeric distribution. His vWF:ristocetin cofactor assay was < 20. The Bethesda assay (factor VIII inhibitor screening) was negative for an inhibitor. Circulating anticoagulant screening (mixing study) failed to correct his PTT which suggested the possibility of an inhibitor or lupus anticoagulant. Although he was positive for lupus anticoagulant (anti-LA 67), dilute Russell viper venom time (DRVVT) ratio was 1. Thus, we ruled out antiphospholipid antibody syndrome and diagnosed the patient's condition as acquired von Willebrand factor deficiency. The patient was resuscitated with 3 units of packed red blood cells and prothrombin complex concentrate. He underwent factor VIII 50% replacement, one dose of haemate-p (3976 units), one cycle of rituximab, and three weeks of oral steroids. He also received two cycles of IVIg which normalized vWF antigen or factor VIII activity. His aPTT was 34.9 s, factor VIII activity was 114%, von Willebrand factor antigen was 95%, and vWF:ristocetin cofactor assay was 81%.

Evaluation of underlying etiology showed decreased albumin and IgG with normal IgA and IgM on serum electrophoresis. Immunofluorescence showed a band in IgG lambda suggestive of an early monoclonal protein (0.34 g/dL of the total 0.68 g/dL of protein in the gamma region). Kappa/lambda ratio was 0.70 (normal reference range 0.26–1.65). He was diagnosed with monoclonal gammopathy of unknown significance. Bone marrow biopsy to rule out myeloma was not done. CT scans of head, chest, abdomen, and pelvis done were negative for lytic bone lesions or lymphadenopathy. He had normal renal function and normal serum calcium. Iron profile showed ferritin of 925 ng/ml (27–300 ng/dl), iron of 25 mcg/dl (40–175 mcg/dl), and transferrin of 125 mg/dl (193–378 mg/dl). Ferritin was most likely elevated from blood transfusions. Folate and vitamin B12 were normal. ANA, hepatitis B, and hepatitis C were negative. Thyroid function test was normal. Echocardiogram showed mild aortic stenosis. He was discharged on 40 mg prednisone daily after bleeding was controlled. His hemoglobin level on discharge was 9.8 gm/dl.

On follow-up after a month on prednisone, his aPTT was 34.9, factor VIII activity was 114%, von Willebrand factor antigen was 95%, and vWF:ristocetin cofactor assay was 81%. His hemoglobin was 11.8 g/dl. Repeat iron studies were not done.

## 3. Discussion

Acquired vWS is a rare condition [5]. It is associated with a multitude of conditions, including lymphoproliferative disorders (most common: 48%), myeloproliferative disorders (chronic granulocytic leukemia, essential thrombocythemia, and polycythemia vera), neoplasms (Wilms tumor), immunological disorders, cardiovascular diseases, hypothyroidism, hemoglobinopathies, drugs (valproate, ciprofloxacin, and hydroxyethyl starch), and infections [1, 2, 4–7]. Among lymphoproliferative disorders, MGUS is the most commonly associated with AvWD, including in 23% of registered patients [1]. Other lymphoproliferative disorders associated with acquired vWD are MM, Waldenstrom macroglobulinemia, CLL, HCL, NHL, and lymphosarcoma [3]. There are two proposed mechanisms for acquired vWD. Immune mechanism is believed to be the responsible mechanism in patients with lymphoproliferative or autoimmune diseases. Nonspecific antibodies bind to vWF; this complex gets cleared by FC-bearing cells or autoantibodies directed against the vWF [5, 8]. In MGUS, as in our patient, paraproteins bind to vWF resulting in accelerated clearance and low circulating levels [2]. Immune-mediated cause most likely mediates the disease process in our patient, as he had significant response to IVIg. Nonimmune-mediated mechanisms are thought to be due to loss of large vWF multimers under high shear stress conditions (congenital heart defects, aortic stenosis, artificial heart valves, or left ventricular assist device), absorption of vWF onto tumor cells, decreased synthesis (hypothyroidism and valproic acid) or release from endothelial cells, or increased proteolysis of vWF (ciprofloxacin) [2, 4, 5].

Presentation in acquired vWD is similar to congenital vWD. However, AvWD presents later in life with no previous or family history of bleeding [9, 10]. Routine recommended tests in acquired von Willebrand disease are vWF:Ag assay, vWF:Ag activity assay, and ristocetin cofactor assay (vWF:RCo/Ag ratio) (normal = 1) [2, 7]. Among them, ristocetin cofactor assay is the recommended test [7]. vWF:RCo/Ag ratio < 0.6–0.7 indicates inhibitory antibodies or a selective loss or decrease in high molecular weight multimers [2]. Circulating inhibitors are rarely detected in this condition [4]. vWF multimer analysis is the gold standard test. It is particularly useful if other tests are negative [2]. A multimeric pattern with decreased levels of high molecular weight vWF multimers is seen because the inhibitors tend to attack the large multimers of vWF. Serum protein electrophoresis or immunofluorescence tests and thyroid function test can be done to establish etiology [4]. Congenital vWD and AvWD can be differentiated by measurement of vWF propeptide (vWFAg II). vWF propeptide is a marker of vWF synthesis. Since AvWD is associated with clearance of vWF, vWF propeptide will be normal [4].

## 4. Management

Management is based on correction of acute bleeding episode and treatment of associated underlying conditions. The acute bleeding can be controlled by infusion of cryoprecipitate, vWF-containing factor VIII (FVIII) concentrates (haemate-p), or desmopressin. This will result in immediate and transient rise in serum FVIII-vWF complex levels [4]. IVIg can correct vWF activity for 2–3 weeks [2]. IVIg is the preferred to minimize bleeding risk during elective procedures [1]. A combination of IVIg, vWF-containing factor VIII (FVIII) concentrates (haemate-p), and desmopressin is effective in some patients [5]. FVIII and vWF levels return to baseline within 2 hours of transfusion of haemate-p [11]. In patients with MGUS, steroids and rituximab have been used to treat AvWD [5]. Rituximab, which is effective in acquired hemophilia, has been used in treatment for a few cases. However, it has been reported to be ineffective in them [12]. Management of aortic stenosis, hypothyroidism, and discontinuation of medications has resulted in correction of the bleeding disorder [2].

## 5. Conclusion

AvWD should be considered in cases with abnormal bleeding and prolonged PTT. It can be asymptomatic in mild conditions for decades [2]. Further workup for an etiology is important as treatment of the underlying cause can prevent future bleeding episodes.

## Figures and Tables

**Table 1:** Lab tests done during presentation, treatment, and a month after treatment.

Lab test	Presentation	During treatment (3 days)	1 month after treatment	Reference range
INR	1.1	N/A	1.1	0.9–1.1
PTT (sec)	48	N/A	34.9	23–34 s
Factor VIII activity (%)	12	33	114	50–160%
Factor IX activity (%)	166	N/A	N/A	60–150%
Factor XI activity	98	N/A	N/A	50–150%
Factor XII activity	54	N/A	N/A	50–150%
vWF antigen (%)	6	10	95	60–150%
Ristocetin cofactor assay (%)	< 20	< 20	81	50–160%
vWF multimer analysis	Normal	N/A	N/A	Normal multimeric distribution
Bethesda assay	0	N/A	N/A	0

INR: international normalized ratio; PTT: partial thromboplastin time; vWF: von Willebrand factor; N/A: not available.
